# Impact of rehabilitation unit-based physical activity therapy versus symptomatic supportive treatment on older patients with advanced cancer: a non-randomized controlled study

**DOI:** 10.1007/s00520-024-08701-1

**Published:** 2024-07-15

**Authors:** Xiaoqiong Lu, Shubao Wei, Benzi Liang, Cheng Huang, Weiwei Meng, Xiaojing Zhang, Xiuqiong Chen

**Affiliations:** Department of Pain Rehabilitation, Jiangbin Hospital of Guangxi Zhuang Autonomous Region, 85 Hedi Road, Nanning, 530000 Guangxi Province China

**Keywords:** Advanced cancer, Older patients, Physical activity therapy, Rehabilitation

## Abstract

**Objectives:**

Relatively few studies have investigated the effects of rehabilitation-based physical activity therapy as a treatment for older patients with advanced cancer. This study evaluated the effects of individualized precise and structured exercise interventions, prescribed by a rehabilitation physician, on fatigue, quality of life (QOL), and physical activity in older patients with advanced cancer.

**Methods:**

After admission to the rehabilitation department, older cancer patients were divided into groups receiving conventional symptomatic supportive therapy (SST) or physical activity therapy plus conventional symptomatic supportive therapy (PAT). The SST group was given symptomatic supportive treatment, exercised on their own, and were observed at home after their symptoms improved. The PAT group was required to implement physical exercise along with SST, involving 30 min of moderate-intensity exercise per day and 5 days per week, and were discharged after 4 weeks and instructed to continue to exercise outside the hospital. Cancer-related fatigue (CRF) at 4 and 8 weeks was the primary endpoint of the study, while the secondary endpoints included patients’ QOL, physical activity, and exercise adherence rate.

**Results:**

Sixty-five patients were included; 37 (56.92%) chose to enter the PAT group, and 28 (43.08%) chose to enter the SST group. After 4 and 8 weeks of treatment, CRF relief and QOL improvement were significantly better in the PAT group than in the SST group (*p* < 0.05), whereas global health status did not differ between the two treatment groups (T1: *p* = 0.84; T2: *p* = 0.92). Mild physical activity significantly increased for the PAT group at T1 and T2 (T1: *p* = 0.03; T2: *p* = 0.005). At the T2 time point, the PAT group exhibited a higher level of participation in moderate-intensity physical activities as well as a higher total leisure activity score (*p* < 0.05). Thirty-three patients (94.29%) completed the PAT exercise program during hospitalization. Only four (12.12%) patients achieved moderate-intensity exercise, while the other 29 (87.88%) patients were able to continue exercising after their exercise intensity was decreased.

**Conclusions:**

Implementation of precise and individualized exercise interventions, prescribed by the rehabilitation team, can lead to the reduction of CRF and improvement of QOL, and change in behavior related to physical activity.

## Introduction

Morbidity and mortality due to cancer increase markedly with age [1]. By 2030, one-third of all patients with cancer are predicted to be aged over 70 years [2]. In addition, with the development of medicine and advances in treatment, the survival rates among patients with cancers, such as lung cancer, breast cancer, and nasopharyngeal cancer, have continued to increase [3], and patients increasingly survive cancer until older age (≥ 65 years). It is expected that, by 2040, about 73% of patients with cancer will be over 65 years old [4, 5]. Therefore, as the number of older adults increases and medical technology continues to advance, the number of older patients with cancer will continue to increase, imposing a heavy burden on and a great challenge to healthcare systems in all countries.

In older individuals, including older patients with cancer, the functions of various organs in the body, including cardiopulmonary function and muscle strength, deteriorate with increasing age [6]. Older patients with cancer may also face problems such as poor health, functional limitations, and comorbidities [7], which result in more severe cancer-related fatigue (CRF) and somatic and psychological dysfunction during the diagnosis and treatment of cancer than do the younger population. These issues may last for a long time and have a serious impact on the patient’s quality of life (QOL). In a survey of cancer survivors, in which the proportion of older patients with cancer was as high as 48.5%, 38.2% reported having physical and psychological disorders and CRF [8]. These complex and multiple disorders and symptoms of discomfort seriously affect QOL. Therefore, the goal of treatment of older patients with cancer should not only be to prolong survival time, but should also consider the patient’s needs in terms of QOL and should provide comprehensive rehabilitation treatment and support.

In recent years, several guidelines [9–13] have suggested that physical activity therapy (PAT) can reduce CRF, improve QOL and physical fitness, and restore physical function in patients with cancer. It has also been shown [14, 15] that PAT can be an effective means to help patients maintain body weight, can prevent or improve cachexia, has few side effects, and is an effective and safe nonpharmacological therapy. However, older patients with cancer may face a number of challenges when participating in a PAT-based clinical trial and, therefore, are underrepresented in previous clinical trials [16–19]. Although the 2016 American Study Group on Cancer and Aging made some recommendations for clinical trials on PAT among older patients with cancer [20], relatively little evidence from current guidelines and studies exists for this population, particularly for frail older patients who have finished treatment or refused antineoplastic therapy, and this population has also received less attention from oncologists.

Home exercises, such as cycling and yoga, supervised by nursing staff or exercise professionals, are a common research approach in the field [21–23]. Furthermore, with the development of rehabilitation medicine, the concept of oncology rehabilitation has continuously been inculcated, and it has become more common for cancer patients, particularly older patients, to undergo rehabilitation training in the rehabilitation department, as an interdisciplinary approach. However, research findings on precise, individualized, and structured physical activity interventions during hospitalization, under the auspices of a professional rehabilitation team, including rehabilitation physicians and therapists in the rehabilitation department, are lacking.

We sought to provide more effective rehabilitation programs for older patients, in order that they can maintain good body function and improve their health status and QOL during their limited life. Thus, in this study, we investigated the effect of precise and individualized exercise training in older patients with advanced cancer, under the supervision of a rehabilitation team. Specifically, this study aimed to determine whether CRF and QOL could be improved in older patients with advanced cancer using precise and individualized PAT prescribed by a rehabilitation team.

## Methods

### Study design

This was a two-group, prospective, non-randomized controlled study. After hospitalization in the rehabilitation department, older patients with advanced cancer were divided into the conventional symptomatic supportive treatment (SST group) and the PAT with conventional SST (PAT group) groups, as elected by the patient.

This study was approved by the Medical Ethics Committee of the research institution (registration number:2024–008). Written informed consent was obtained from all patients participating in the study.

### Inclusion and exclusion standards

We included patients aged ≥ 65 years with advanced tumors, diagnosed as malignant, with metastasis or progression, by pathological examination; patients with a Karnofsky functional status score (KPS) ≥ 60; patients with a life expectancy of 3 months or longer; and patients for whom intravenous chemotherapy or intravenous targeted drugs was not planned (because the hospitalization unit was a rehabilitation department).

The exclusion criteria were as follows: severe cognitive impairment or emotional instability; communication disorders; and comorbidities, such as heart failure, respiratory failure, and other diseases that could seriously affect the patient’s activities.

### Intervention

#### SST group

According to the symptoms of the patients and the relevant examination results, appropriate SST (such as oral or intravenous nutritional support, pain control, laxatives, and anti-insomnia, anxiety, and depression medications) was provided. At the same time, patients were told that physical exercise could improve their symptoms of physical dysfunction and psychological disorders and reduce CRF and discomfort, and they were encouraged to exercise according to their own interests. After their symptoms improved, the patient could be discharged from the hospital (length of hospitalization ≤ 4 weeks) and continue to take oral medication for symptomatic treatment outside the hospital. If a patient’s condition deteriorated during hospitalization and the patient required long-term hospitalization, the patient was excluded from the analysis.

#### PAT group

For PAT, rehabilitation physicians develop individualized exercise prescriptions for each patient, based on their condition and physical status, including the type, intensity, frequency, and duration of physical activity. During the PAT process, a therapist supervised and guided the patient to ensure the accuracy and safety of performing the workout. The physician and therapist also adjusted the exercise prescription according to the patient’s physical condition and level of fatigue, to achieve the best therapeutic effect.

The patients were required to perform PAT while also undergoing SST. According to international exercise guidelines for oncology patients, the total weekly PAT time should not be less than 120–150 min [9, 10, 24]. The duration and frequency of PAT in this study was 30 min of moderate-intensity exercise per day, with 5 min of warm-up and relaxation before and after the exercise, and 5 days of exercise per week, including aerobic exercise, muscular endurance training, and balance training. Aerobic exercises were performed at a moderate intensity and adjusted according to the patient’s physical tolerance. Muscular endurance training targets six major muscle groups (hamstrings, quadriceps, gluteus maximus, deltoids, biceps, and triceps). The PAT goal was 50–75% of the maximum heart rate, for 30 min. Balance training was used to complement aerobic and muscular endurance training with the aim of improving the patient’s ability to perform daily activities. Specific PAT prescriptions were formulated by a rehabilitation physician according to the patient’s condition, and the therapist supervised and instructed the patient. During the treatment process, the rehabilitation physician adjusted the exercise prescription according to the patient’s physical condition and CRF level. The patient was discharged after approximately 4 weeks of hospitalization. Before discharge, the patient was taught some simple home PAT programs and encouraged to persist in light-to-moderate intensity PAT for a long period, and the caregiver was advised to supervise the patient’s workout.

### Primary and other outcome measures

The primary study outcome was improvement in CRF, and the secondary study endpoints included changes in QOL, physical activity, and adherence rate. Each participant was assessed at baseline (T0), after 4 weeks of treatment (T1), and at the outpatient follow-up at week 8 (T2). Fatigue was assessed using the Piper fatigue scale, which represents the severity of fatigue using specific scores, with 0 indicating no fatigue, 1–3 indicating mild, 4–6 indicating moderate, and 7–10 indicating severe fatigue. The QOL was primarily assessed using the European Organization for Research and Treatment of Cancer (EORTC) QLQ-C30 questionnaire. This validated cancer-specific tool [25, 26] assesses physical, role, cognitive, emotional, and social domains of functioning; nine symptoms, including fatigue, pain, nausea and vomiting, dyspnea, loss of appetite, insomnia, constipation, diarrhea, financial difficulties; and global health status. Only the five domains of functioning, global health status, and fatigue symptoms were assessed in this study. The EORTC QLQ-C30 was analyzed according to the EORTC guidelines [27]. Higher scores for the functioning and global health status domains suggest better functional status and quality of life, whereas higher scores for the symptom domain indicate more symptoms or problems and a worse QOL. Physical activity was assessed using the Godin Leisure-Time Exercise Questionnaire [28] to assess the mean frequency and duration of mild, moderate, and strenuous exercise. The instrument is considered a reliable measure of recent physical activity and has been extensively validated in diverse populations [29]. Participants in the exercise group were instructed to only engage in physical activity outside of the intervention. The adherence rate was defined as the proportion of participants in the PAT group who successfully completed 120–150 min of physical activity per week in weeks 1 to 4 of the hospitalization study.

### Statistical analysis

The sample size calculation was based on the primary endpoint and the physical fatigue of the Piper fatigue scale at T1, as we considered that the effect of exercise on CRF would be the strongest at that time point. In order to detect a 3 point reduction on the physical fatigue between intervention group and control group (i.e., reduction of 30% of the reference), the sample size calculation was based on a two-sided alpha of 0.05, 90% power, SD of 3, and with a hypothetical correlation coefficient between measures of 0.2 [30]. The sample size was estimated to be 26 patients per arm in PASS15. Considering a 20% loss to follow-up, we aimed to enroll 33 patients per group overall in the trial.

Statistical data analysis was carried out using descriptive statistical methods using IBM SPSS Statistics v25 software (IBM SPSS Inc., Armonk, NY, USA). Data cleaning was performed to discover outliers, and if any were found, they were corrected with reference to the original records. Continuous variables were presented as the mean and standard deviation, and count data were presented as percentages. The evolution of the CRF, QOL, and physical activity scores over time was assessed using linear mixed models. Random intercepts and slopes were included to account for time effects. The interaction terms were also considered categorical, and ordinal variables were compared using the chi-squared tests. Continuous variables were presented as the mean and standard deviation (SD) and were compared using the *T* test.

The model coefficients were estimated using maximum likelihood. Statistical significance was set at *p* < 0.05.

## Results

**Fig. 1:**
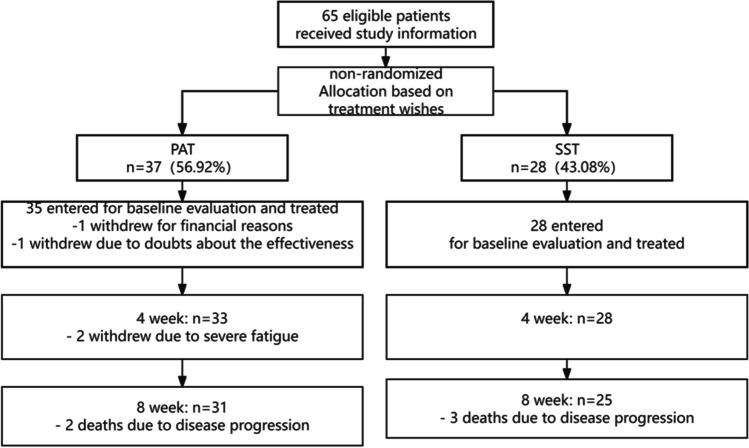
CONSORT diagram of the stud

From March 2020 to March 2023, 65 older patients (aged ≥ 65 years) with advanced cancers, including lung, breast, gastric, esophageal, colon, and cervical cancers, were enrolled. Thirty-seven (56.92%) patients chose to utilize PAT, and 28 (43.08%) patients chose to utilize SST. Common symptoms in patients are centered on pain, decreased appetite, sleep disturbances, and stool abnormalities. Mild dependence is predominant in the ability of daily living(ADL), with more moderate dependence on assistance when instrumental ability of daily living(IADL) are required. In terms of nutritional status, it is noteworthy that about half of the patients were cachectic. PAT group was given conventional symptomatic supportive treatment and physiotherapist supervised and guided personalized exercise, and SST group was only received conventional symptomatic supportive treatment and exercise according to their own interests (Table 1). After enrollment and before completion of the baseline assessment, two patients in the PAT group withdrew because of the increased financial burden of PAT and doubts about the efficacy of the treatment (Fig. 1). At baseline assessment, the median age, KPS score, and other basic characteristics of the patients in the two groups were similar (Table 2). Of the 35 patients that entered the PAT group, two (5.71%) withdrew (one each after the 1st and 2nd week after the start of treatment) because they were too tired to continue. Thirty-three patients insisted on completing the PAT during hospitalization; the adherence rate was 94.29%, but only four (12.12%) patients reached moderate intensity, while the remaining 29 (87.88%) patients felt that moderate intensity was too strenuous, but were able to continue PAT after the intensity was reduced. At the 8-week follow-up, two patients in the PAT group and three patients in the SST group had died due to disease progression.

**Table 1:** The contents of PAT and SST

	Weeks 1 to 4 (hospitalization)	Weeks 5 to 8 (outside the hospital)
PAT	Conventional symptomatic supportive treatment + physiotherapist supervised and guided personalized exercise	Take oral medication and simple home PAT programs
SST	Conventional symptomatic supportive treatment and exercise according to their own interests	Take oral medication

**Table 2:** Baseline characteristics of patients with the intention of treating the population with the intention of treating the population

Characteristic	PAT	SST	*x*^2^	*p*
	37	28		
Sex *N*(%)				
Male	16 (43.24)	11 (39.29)	1.55	0.24
Female	21 (56.76)	17 (60.71)		
Age				
Mean year(SD)	75.83 (7.71)	72.46 (7.50)	*t* = 1.64	0.4
Age group				
65 year ~ 75 year *N*(%)	16 (43.24)	11(39.29)	1.55	0.24
≥ 75 year *N*(%)	21 (56.76)	17 (60.71)		
Diagnosis *N* (%)				
Lung cancer	8 (21.62)	6 (21.42)	5.36	0.62
Breast cancer	5 (13.51)	4 (14.29)		
Stomach, esophagus cancer	3 (8.10)	2 (7.14)		
Colorectal cancer	5 (13.51)	4 (14.29)		
Cervical cancer	5 (13.51)	4 (14.29)		
Ovarian cancer	3 (8.10)	2 (7.14)		
Prostate cancer	3 (8.10)	2 (7.14)		
Other tumors	5 (13.51)	4 (14.29)		
Cancer treatment *N* (%)				
Surgery	22 (59.46)	17 (60.71)	0.01	0.92
Chemotherapy	34 (91.89)	26 (92.85)	0.02	0.89
Targeted therapy	16 (43.24)	12 (42.86)	0.001	0.98
Radiotherapy	18 (48.65)	12 (42.86)	0.22	0.64
Physical symptoms *N* (%)				
Pain	19 (51.35)	13 (46.42)	0.16	0.69
Gastrointestinal symptoms	26 (70.27)	19 (67.86)	0.04	0.84
Dyspnea	4 (10.81)	5 (17.86)	0.65	0.42
Sleep disturbance	23 (62.16)	16 (57.14)	0.17	0.68
Abnormal stool	17 (45.95)	13 (46.43)	0.001	0.97
Karnofsky (KPS) performance status *N* (%)				
< 60	0 (0)	0 (0)	0.85	0.66
60	6 (16.21)	7 (25.00)		
70	15 (40.54)	11 (39.29)		
80	16 (43.24)	10 (35.71)		
> 80	0 (0)	0 (0)		
ADL mean points(SD)	73.51 (7.98)	75.54 (7.62)	*t* = − 1.03	0.31
IADL *N* (%)				
8 points (normal)	2 (5.40)	1 (3.57)	0.14	0.93
6–7 points (mild dependence)	15 (40.54)	12 (42.86)		
3–5 points (moderate dependence)	20 (54.05)	15 (53.57)		
BMI (kg/m^2^) *N*(%)				
≥ 18.5	20 (54.05)	12 (42.86)	0.80	0.37
< 18.5	17 (45.95)	16(57.14)		
Economic situation *N* (%)				
Retirement pay	19 (51.35)	13 (46.43)	0.16	0.69
No retirement pay	18 (48.65)	15 (53.57)		

At the baseline assessment, both groups had similar levels of CRF and were moderately fatigued (*p* > 0.05). After 4 weeks of treatment, the PAT group showed significantly greater relief of physical, emotional, sensory, and cognitive fatigue than did the SST group (*p* < 0.05). The PAT group still showed significantly less fatigue in all domains than the SST group at week 8 (*p* < 0.05) (Table 3).

**Table 3:** Fatigue of the Piper fatigue scale, quality of life (EORTC QLQ-C30), and physical activity with the intention of treating the population

		Baseline(T0)			End of 4 week (T1)			End of 8 week (T2)		
Fatigue: Piper fatigue scale		Mean (SD)	Est (95% CI)	*P*	Mean (SD)	Est. (95% CI)	*P*	Mean (SD)	Est.(95% CI)	*P*
Physical fatigue	PAT	5.47 (0.11)	0.15 (− 0.17 ~ 0.45)	0.37	4.68 (0.12)	− 0.51 (− 0.82 ~ − 0.20)	0.001	4.53 (0.09)	− 0.86 (− 0.17 ~ − 0.55)	< 0.001
	SST	5.33 (0.12)			5.12 (0.13)			5.38 (0.10)		
Emotional fatigue	PAT	4.53 (0.07)	0.08 (− 0.09 ~ 0.25)	0.34	3.9 (0.06)	− 0.35 (− 0.50 ~ 0.18)	< 0.001	4.02 (0.05)	− 0.38 (− 0.52 ~ 0.23)	< 0.001
	SST	4.45 (0.07)			4.33 (0.07)			4.40 (0.05)		
Sensory fatigue	PAT	5.21 (0.10)	0.05 (− 0.24 ~ 0.35)	0.73	4.41 (0.10)	− 0.73 (− 1.07 ~ − 0.43)	< 0.001	4.89 (0.10)	− 0.34(− 0.63 ~ 0.04)	0.03
	SST	5.16 (0.11)			5.14 (0.11)			5.23 (0.11)		
Cognitive fatigue	PAT	5.23 (0.08)	0.10 (− 0.31 ~ 0.07)	0.23	4.37 (0.06)	− 0.41 (0.60 ~ 0.22)	< 0.001	4.25 (0.05)	− 0.67 (0.86 ~ 0.48)	< 0.001
	SST	5.35 (0.08)			4.78 (0.07)			4.92 (0.06)		
EORTC QLQ-C30										
Global health status	PAT	46.54 (1.73)	0.29 (− 4.92 ~ 5.51)	0.91	53.23 (1.66)	− 0.49 (− 5.33 ~ 4.35)	0.84	50.92 (1.40)	− 0.22 (− 5.06 ~ 4.62)	0.92
	SST	46.71 (1.93)			53.71 (1.84)			51.14 (1.66)		
Physical functioning	PAT	61.29 (1.74)	1.66 (− 1.65 ~ 4.97)	0.32	73.06 (1.74)	5.87 (0.65 ~ 11.08)	0.02	68.87 (1.74)	5.07 (− 0.14 ~ 10.29)	0.56
	SST	61.00 (1.94)			67.20 (1.94)			63.8 (1.94)		
Role functioning	PAT	57.66 (1.35)	0.52 (− 3.43 ~ 4.46)	0.8	74.60 (0.72)	10.6 (8.43 ~ 12.77)	< 0.001	70.57 (1.18)	7.06 (3.52 ~ 10.61)	< 0.001
	SST	56.00 (1.51)			64.00 (0.81)			63.50 (1.32)		
Cognitive functioning	PAT	64.52 (1.58)	− 0.85 (− 3.71 ~ 2.02)	0.56	84.27 (1.05)	10.27 (7.13 ~ 13.42)	< 0.001	79.03 (1.32)	8.53 (4.59 ~ 12.48)	< 0.001
	SST	64.00 (1.76)			74.00 (1.17)			70.50 (1.46)		
Emotional functioning	PAT	69.15 (1.22)	3.48 (− 0.84 ~ 7.81)	0.11	77.82 (0.68)	3.32 (1.30 ~ 5.35)	0.002	75.61 (0.93)	5.61 (2.83 ~ 8.38)	< 0.001
	SST	70.00 (1.36)			74.50 (0.75)			70.00 (1.03)		
Social functioning	PAT	35.48 (1.44)	− 3.17 (− 8.01 ~ 1.67)	0.2	60.08 (1.26)	15.68 (11.80 ~ 19.35)	< 0.001	50.00 (1.62)	6.5 (1.63 ~ 11.37)	0.01
	SST	32.00 (1.61)			44.50 (1.40)			43.50 (1.81)		
Fatigue symptom	PAT	52.42 (1.34)	− 3.10 (− 6.98 ~ 0.78)	0.12	38.44 (1.34)	− 10.56 (− 14.44– − 6.68	< 0.001	43.01 (1.34)	− 6.66 (− 10.65– − 2.67)	0.001
	SST	55.52 (1.31)			49 (1.46)			49.67 (1.34)		
Physical activity(min/week)										
Moderate physical activity	PAT	42.58 (3.39)	1.38 (− 8.75 ~ 11.51)	0.79	47.10 (3.50)	5.7 (− 4.43 ~ 5.82)	0.26	46.77 (3.21)	5.97 (− 4.16 ~ 16.11)	0.24
	SST	41.20 (3.94)			41.40 (3.89)			40.80 (3.58)		
Mild physical activity	PAT	40.54 (2.54)	− 0.49 (− 8.09 ~ 7.11)	0.9	50.16 (2.82)	8.12 (0.52 ~ 15.72)	0.03	53.42 (2.47)	10.94 (3.34 ~ 18.54)	0.005
	SST	41.04 (2.82)			44.04 (3.14)			42.48 (2.75)		
Total leisure activity score	PAT	83.13 (5.72)	0.89 (− 16.28 ~ 18.05)	0.918	99.26 (5.92)	13.82 (− 3.96 ~ − 31.56)	0.125	100.19 (5.33)	16.91 (− 0.93 ~ 32.89)	0..038
	SST	82.24 (6.37)			85.44 (6.60)			83.28 (5.93)		

Lower mean scores represent less fatigue and functional impairments. Higher mean scores represent a better quality of life or functioning. *p* < 0.05 indicates significance for the outcome; *CI* confidence interval, *Est*. estimated mean difference, *ORTC-QLQ-C30* European Organization for Research and Treatment of Cancer-Quality of Life Questionnaire-Core 30, *PAT* physical activity therapy, *SST* symptomatic supportive treatment group

Functional domain scores of the physical body were similar in QOL of both groups (*p* > 0.05) (Table 3). After 4 weeks of treatment, the physical, role, cognitive, emotional, and social functional domain scores of the PAT group were significantly higher than those of the SST group (*p* < 0.05). At week 8, except for the physical functional domains, which were not significantly different between the two groups (*p* = 0.56), the role, cognitive, emotional, and social functional domain scores of the PAT group remained higher than those of the SST group (*p* < 0.05). In contrast, the global health status score was not significantly different between the two treatment groups, regardless of the time points (*p* = 0.84 at T1; *p* = 0.92 at T2). In terms of fatigue symptoms, PAT was associated with less fatigue than SST at both time points after treatment, and the difference was significant (T1, *p* < 0.001; T2, *p* = 0.001).

Both groups exhibited similar physical activity levels at the baseline (*p* > 0.05) (Table 3). There was no vigorous physical activity in both groups during hospitalization and follow-up outside the hospital. Mild physical activity significantly increased for the PAT group at T1 and T2 compared to the baseline (T1, *p* = 0.03; T2, *p* = 0.005). The PAT group exhibited a higher level of participation in moderate-intensity physical activities and a higher total leisure activity score than the SST group at the T2 time point (*p* < 0.05).

## Discussion

Here, we investigated the impact of optimal symptomatic supportive care in the rehabilitation department, along with “precision” and individualized exercise performed under the auspices of a rehabilitation team on the CRF, QOL, and physical activity of older patients with advanced cancer. Patients in the PAT group showed more significant improvements in physical, emotional, sensory, and cognitive fatigue states after individualized exercise at the T1 and T2 points compared to the SST group. The PAT group showed a statistically significant advantage in QOL over the SST group at both time points in the subdomains of function and fatigue symptoms, except for improvements in physical function at T2, which were essentially like those of the SST group. This finding is consistent with the findings of Carayol et al. [31] whose study examined the effects of exercise on CR and QOL in patients with breast cancer and indicated that exercise was effective in alleviating CRF and enhancing patients’ QOL.

The PAT group had higher total leisure activity scores than the SST group at week 8. Furthermore, it demonstrated longer activity times than the SST group in mild activity at T1 and T2, although the difference between the two groups was insignificant in terms of vigorous and moderate physical activity. This agrees with another study showing that an exercise program might result in changes in participant physical activity that persist well after the end of the program for patients with breast cancer [29].

We demonstrated that the PAT program provided rapid relief of CRF and improved patients’ QOL, and also observed changes in the participants’ behavior related to physical activity. After intensive training under the supervision of a rehabilitation therapist, patients were able to continue to exercise even after they were discharged from the hospital, which continued to have a positive impact on their CRF, QOL, and physical activity.

However, Arrieta et al. [32] in a large randomized controlled study on the effects of PAT on physical functioning in older patients with cancer, showed that personalized physical activity advice did not reduce the decline in physical functioning after 1 year. Their study was based on only personalized exercise advice, provided to patients over the phone, without specific supervision. Multiple studies [23, 33–36] have shown that supervised PAT is superior to unsupervised PAT. In our study, the patients’ exercise prescription was developed by a rehabilitation physician and was adjusted according to the patient’s condition, and the patients were supervised and instructed throughout the PAT process, which may explain the differences in findings from those of Arrieta et al. In addition, the assessment times in this study were 4 and 8 weeks after treatment, which is a short treatment period, whereas the study by Arrieta et al. conducted assessments after 1 year. Therefore, the long-term effects could not be determined in the present study, indicating that further research is needed.

However, although the differences between the two groups were statistically significant before and after treatment in all functional domains of both CRF and QOL, the degree of reduction and improvement was not obvious. In particular, the difference between the two groups in global health status in terms of QOL was not significant at either T1 or T2. This discrepancy may be related to the characteristics of the enrolled patients. The population included in this study was older and may have had continuous cancer progression and a high level of CRF. Most patients could only tolerate low-intensity PAT. Moreover, the duration of hospitalization and follow-up of the patients was relatively short. Recommendations issued by the American College of Sports Medicine (ACSM) on exercise for cancer patients include at least 150 min of moderate-intensity aerobic exercise per week or 30 min of vigorous aerobic exercise 3 days per week [37]. Other studies have also shown that moderate-intensity aerobic training conducted three times per week for at least 12 weeks significantly reduces CRF and improves QOL [38–40]. The patients in this study did not meet these criteria, neither in terms of exercise intensity nor duration; therefore, the program had some efficacy, albeit not particularly significant. Future research may consider increasing the length of inpatient rehabilitation and extending the follow-up time.

Exercise is challenging for older patients with cancer, and adherence to long-term exercise is even more difficult. In addition to the age-related decline in health and fatigue, they may also be affected by other physical limitations and comorbidities [41]. In addition, lack of self-discipline and skepticism about the effectiveness of PAT are the main reasons why patients have difficulty maintaining a long-term PAT program. PAT interventions in previous studies usually consisted of home-directed exercises, based on patients’ interests and physical condition, and included walking, yoga, bicycling, and muscle strength training [23, 42, 43]. However, PAT interventions are usually not guided or supervised by a rehabilitation physician or therapist; thus, patients may have difficulty maintaining long-term exercise habits. In one study of a home-based self-guided exercise program for breast cancer patients, the adherence rate to exercise was only 36% [44], while it was 59.7% in another study of home-based walking exercises for lung cancer patients [45]. In our study, in addition to the supervision, guidance, and encouragement of the rehabilitation therapist during the PAT process, patients were repeatedly informed by the rehabilitation physician about the possibility of malignant disease, physical dysfunction, and disability during the course of cancer, and were informed that PAT could prevent the rapid loss of physical function and improve the QOL to the maximum extent possible in a limited lifespan, which increased patients’ knowledge about disease prognosis. This may explain the high adherence rate (94.29%) observed in the present study.

This study had some limitations. First, the study was a non-randomized controlled study with a small sample size. Second, the enrolled cases included patients with different types of advanced cancer, rather than those with the same type of cancer. Further research should focus on groups of patients with specific types of advanced cancer. Finally, due to the limited hospitalization time, the duration of in-hospital intervention was only 4 weeks, which prevented us from evaluating the effect of long-term PAT in the participants.

This study also had several strengths. The population of this study included older patients with advanced cancer, which comprised a relatively small group of patients in previous studies. We found no adverse effects related to age or treatment during the course of treatment. In addition, this study was based on an intervention completed with the participation of rehabilitation physicians and therapists in the rehabilitation department, which is a part of oncology rehabilitation. Oncology rehabilitation is a relatively new field in palliative care, although it is now more common for oncology patients to visit the rehabilitation department for exercise training in an interdisciplinary approach. Nevertheless, few reports of related studies have been published to date. Therefore, this study can be regarded as an early exploration of this field, providing a basis for active development and improvement of related research and practical activities in the field of oncological rehabilitation.

## Conclusion

This study shows that, while older patients with cancer are hospitalized in the rehabilitation department for the best symptomatic supportive treatment, the “precise” and personalized PAT conducted under the auspices of a professional rehabilitation team can reduce these patients’ CRF, improve their QOL, and result in a change in behavior related to physical activity. Due to the intensive training provided by the rehabilitation therapists and the improved knowledge of the disease prognosis, even after they are discharged from the hospital, they can continue exercising at home, causing their CRF level to reduce further or at least not worsen significantly, and their QOL to improve further or at least not decline significantly. Based on our findings, further studies can investigate transitioning from rehabilitation provider-based exercise prescriptions after a short period of hospitalization to a combination of home-based exercise supervised by rehabilitation outpatient services.

## Data Availability

No datasets were generated or analysed during the current study.
